# Supplementary material for: Prognostic Associations of the Naples Score for Clinical Outcomes in Patients With Acute Myocardial Infarction: A Systematic Review and a Prognostic Meta‐Analysis
Source: Cardiol Res Pract. 2026 Sep 26;2026:4613242. doi: 10.1155/crp/4613242 (PMC13615453; doi:10.1155/crp/4613242)
Supplement: Supplementary file 1 [file CRP-2026-4613242-s001.docx]

**SUPPLEMENTAL APPENDIX**

**For**

**“Prognostic Value of the Naples Score for Clinical Outcomes in Patients With Acute Myocardial Infarction: A Systematic Review and A Prognostic Meta-Analysis”**

**PRISMA CHECKLIST**

| **Section and Topic** | **Item #** | **Checklist item** | **Location where item is reported** |
| --- | --- | --- | --- |
| **TITLE** | | |  |
| Title | 1 | Identify the report as a systematic review. | Page 1 |
| **ABSTRACT** | | |  |
| Abstract | 2 | See the PRISMA 2020 for Abstracts checklist. | Page 3 |
| **INTRODUCTION** | | |  |
| Rationale | 3 | Describe the rationale for the review in the context of existing knowledge. | Page 4-5 |
| Objectives | 4 | Provide an explicit statement of the objective(s) or question(s) the review addresses. | Page 5 |
| **METHODS** | | |  |
| Eligibility criteria | 5 | Specify the inclusion and exclusion criteria for the review and how studies were grouped for the syntheses. | Page 5 |
| Information sources | 6 | Specify all databases, registers, websites, organisations, reference lists and other sources searched or consulted to identify studies. Specify the date when each source was last searched or consulted. | Page 6 |
| Search strategy | 7 | Present the full search strategies for all databases, registers and websites, including any filters and limits used. | Page 6 |
| Selection process | 8 | Specify the methods used to decide whether a study met the inclusion criteria of the review, including how many reviewers screened each record and each report retrieved, whether they worked independently, and if applicable, details of automation tools used in the process. | Page 5  Figure 2 |
| Data collection process | 9 | Specify the methods used to collect data from reports, including how many reviewers collected data from each report, whether they worked independently, any processes for obtaining or confirming data from study investigators, and if applicable, details of automation tools used in the process. | Page 6 |
| Data items | 10a | List and define all outcomes for which data were sought. Specify whether all results that were compatible with each outcome domain in each study were sought (e.g. for all measures, time points, analyses), and if not, the methods used to decide which results to collect. | Page 7 |
|  | 10b | List and define all other variables for which data were sought (e.g. participant and intervention characteristics, funding sources). Describe any assumptions made about any missing or unclear information. | Page 7  Table 1 |
| Study risk of bias assessment | 11 | Specify the methods used to assess risk of bias in the included studies, including details of the tool(s) used, how many reviewers assessed each study and whether they worked independently, and if applicable, details of automation tools used in the process. | Page 7 |
| Effect measures | 12 | Specify for each outcome the effect measure(s) (e.g. risk ratio, mean difference) used in the synthesis or presentation of results. | Page 8 |
| Synthesis methods | 13a | Describe the processes used to decide which studies were eligible for each synthesis (e.g. tabulating the study intervention characteristics and comparing against the planned groups for each synthesis (item #5)). | Page 8 |
|  | 13b | Describe any methods required to prepare the data for presentation or synthesis, such as handling of missing summary statistics, or data conversions. | Page 8 |
|  | 13c | Describe any methods used to tabulate or visually display results of individual studies and syntheses. | Page 8 |
|  | 13d | Describe any methods used to synthesize results and provide a rationale for the choice(s). If meta-analysis was performed, describe the model(s), method(s) to identify the presence and extent of statistical heterogeneity, and software package(s) used. | Page 8 |
|  | 13e | Describe any methods used to explore possible causes of heterogeneity among study results (e.g. subgroup analysis, meta-regression). | Page 8 |
|  | 13f | Describe any sensitivity analyses conducted to assess robustness of the synthesized results. | Page 8 |
| Reporting bias assessment | 14 | Describe any methods used to assess risk of bias due to missing results in a synthesis (arising from reporting biases). | Page 7  Figure 3 |
| Certainty assessment | 15 | Describe any methods used to assess certainty (or confidence) in the body of evidence for an outcome. | Page 7 |
| **RESULTS** | | |  |
| Study selection | 16a | Describe the results of the search and selection process, from the number of records identified in the search to the number of studies included in the review, ideally using a flow diagram. | Page 8, Figure 1 |
|  | 16b | Cite studies that might appear to meet the inclusion criteria, but which were excluded, and explain why they were excluded. | Page 8 |
| Study characteristics | 17 | Cite each included study and present its characteristics. | Page 9  Table 1 |
| Risk of bias in studies | 18 | Present assessments of risk of bias for each included study. | Page 9-10 |
| Results of individual studies | 19 | For all outcomes, present, for each study: (a) summary statistics for each group (where appropriate) and (b) an effect estimate and its precision (e.g. confidence/credible interval), ideally using structured tables or plots. | Page 10-13 |
| Results of syntheses | 20a | For each synthesis, briefly summarise the characteristics and risk of bias among contributing studies. | Page 13 |
|  | 20b | Present results of all statistical syntheses conducted. If meta-analysis was done, present for each the summary estimate and its precision (e.g. confidence/credible interval) and measures of statistical heterogeneity. If comparing groups, describe the direction of the effect. | Page 10-12 |
|  | 20c | Present results of all investigations of possible causes of heterogeneity among study results. | Page 10-12 |
|  | 20d | Present results of all sensitivity analyses conducted to assess the robustness of the synthesized results. | Page 10-12 |
| Reporting biases | 21 | Present assessments of risk of bias due to missing results (arising from reporting biases) for each synthesis assessed. | Page 13 |
| Certainty of evidence | 22 | Present assessments of certainty (or confidence) in the body of evidence for each outcome assessed. | Page 10-12 |
| **DISCUSSION** | | |  |
| Discussion | 23a | Provide a general interpretation of the results in the context of other evidence. | Page 14-18 |
|  | 23b | Discuss any limitations of the evidence included in the review. | Page 17 |
|  | 23c | Discuss any limitations of the review processes used. | Page 17 |
|  | 23d | Discuss implications of the results for practice, policy, and future research. | Page 14-16 |
| **OTHER INFORMATION** | | |  |
| Registration and protocol | 24a | Provide registration information for the review, including register name and registration number, or state that the review was not registered. | Page 5 |
|  | 24b | Indicate where the review protocol can be accessed, or state that a protocol was not prepared. | Page 5 |
|  | 24c | Describe and explain any amendments to information provided at registration or in the protocol. | Page 5 |
| Support | 25 | Describe sources of financial or non-financial support for the review, and the role of the funders or sponsors in the review. | Page 18 |
| Competing interests | 26 | Declare any competing interests of review authors. | Page 18 |
| Availability of data, code and other materials | 27 | Report which of the following are publicly available and where they can be found: template data collection forms; data extracted from included studies; data used for all analyses; analytic code; any other materials used in the review. | Page 19 |

**SEARCH STRING:**

("Naples Prognostic Score" OR "Naples score" OR NPS OR "Naples prognostic index")

AND

("acute myocardial infarction" OR AMI OR "ST-elevation myocardial infarction" OR STEMI OR

"non-ST elevation myocardial infarction" OR NSTEMI OR "heart attack" OR "myocardial infarct")

AND

(prognosis OR prognostic OR predictive OR outcome OR outcomes OR mortality OR survival OR

"major adverse cardiovascular events" OR MACE OR "clinical outcomes" OR follow-up)

**Supplementary Table S1.** Definitions of outcome measures

| **Outcome Measure** | **Definition** |
| --- | --- |
| 1. **All-cause Mortality** | Death from any cause, regardless of underlying disease or contributing factors, within a specified period of time. |
| 2. **In-hospital Mortality** | Death occurring during a patient’s hospital admission, irrespective of the cause. |
| 3. **No-Reflow Phenomenon** | Inadequate myocardial perfusion at the microvascular level despite successful opening of the epicardial coronary artery after reperfusion therapy. |
| 4. **NOAF Prediction** | Estimation of the risk of developing new-onset atrial fibrillation using clinical, laboratory, or imaging parameters. |

**Supplementary Table S2.** GRADE assessment

| **Using the Naples Prognostic Score as a prognostic value in patients with myocardial infarction** | | | | | |
| --- | --- | --- | --- | --- | --- |
|  | | | | | |
| **Outcomes** | **№ of participants (studies) Follow-up** | **Certainty of the evidence (GRADE)** | **Relative effect (95% CI)** | **Anticipated absolute effects** | |
|  |  |  |  | **Risk in Low NPS group** | **Risk difference in High NPS group** |
| All-cause Mortality | 4367 (4 non-randomised studies) | ⨁⨁◯◯ Low^a,b^ | **OR 3.31 (1.62 to 6.77)** | 81 per 1,000 | **145 more per 1,000**  (from 44 more to 293 more) |
| New-onset atrial fibrillation | (3 non-randomised studies) | ⨁⨁◯◯ Low^a^ | **OR 2.172** (1.628 to 2.899) | 0 per 1,000^c^ | **2 fewer per 1,000** (from 3 fewer to 2 fewer) |
| In-hospital mortality | 10896 (4 non-randomised studies) | ⨁◯◯◯ Very low^a,d,e^ | **OR 1.90** (0.66 to 5.44) | 56 per 1,000 | **45 more per 1,000**  (from 18 fewer to 188 more) |
| No- Reflow | 10898 (3 non-randomised studies) | ⨁⨁◯◯ low^a^ | **OR 1.40** (1.11 to 1.76) | 145 per 1,000 | **47 more per 1,000**  (from 13 more to 85 more) |
| * The risk in the high-NPS group (and its 95% confidence interval) is based on the assumed risk in the low-NPS group and the pooled relative effect (and its 95% CI).  **CI:** confidence interval; **OR:** odds ratio | | | | | |
| **GRADE Working Group grades of evidence** **High certainty:** we are very confident that the true effect lies close to that of the estimate of the effect. **Moderate certainty:** we are moderately confident in the effect estimate: the true effect is likely to be close to the estimate of the effect, but there is a possibility that it is substantially different. **Low certainty:** our confidence in the effect estimate is limited: the true effect may be substantially different from the estimate of the effect. **Very low certainty:** we have very little confidence in the effect estimate: the true effect is likely to be substantially different from the estimate of effect. | | | | | |

**Explanations**

a. Most studies had a moderate risk of bias according to ROBINS-I.

b. The initial heterogeneity was I^2^ = 61.4%. Leave-one-out analysis was performed but heterogeneity remained >50%.

c. Data not reported.

d. The initial heterogeneity was I^2^ = 93.8%. Leave-one-out analysis was performed but heterogeneity remained >50%.

e. Wide confidence interval crossing the null.

| Study | D1 | D2 | D3 | D4 | D5 | D6 | D7 | Overall |
| --- | --- | --- | --- | --- | --- | --- | --- | --- |
| Birdal 2023 | Moderate | Low | Low | Low | Low | Low | Low | Moderate |
| Erdogan 2023 | Moderate | Low | Low | Low | Moderate | Low | Low | Moderate |
| Karakoyun 2023 | Moderate | Low | Low | Low | Low | Low | Low | Moderate |
| Berhan 2025 | Moderate | Low | Low | Low | Low | Low | Low | Moderate |
| Faysal 2025 | Moderate | Low | Low | Low | Moderate | Low | Low | Moderate |
| Gitmez 2025 | Moderate | Low | Low | Low | Low | Low | Low | Moderate |
| Ling 2025 | Moderate | Low | Low | Low | Moderate | Low | Low | Moderate |
| Mehmat 2025 | Moderate | Low | Low | Low | Moderate | Low | Low | Moderate |
| Osken 2025 | Moderate | Low | Low | Low | Low | Low | Low | Moderate |

**Supplementary Table S3**. Robin 1 assessment

**Supplementary Figure S1. Advanced diagnostics and sensitivity analysis for All-cause Mortality**

- **Supplementary Figure S1A:** Leave-one-out sensitivity analysis

- **Supplementary Figure S1B:** Funnel plot for publication bias

- **Supplementary Figure S1C:** Baujat plot
- **Supplementary Figure S1D:** Comprehensive influence diagnostics (Cook’s distance and DFFITS)

**Supplementary Figure S2. Advanced diagnostics and sensitivity analysis for In-hospital Mortality**

- **Supplementary Figure S2A:** Leave-one-out sensitivity analysis

- **Supplementary Figure S2B:** Funnel plot

- **Supplementary Figure S2C:** Baujat plot

- **Supplementary Figure S2D:** Comprehensive influence diagnostics

**Supplementary Figure S3. Advanced diagnostics and sensitivity analysis for No-Reflow Phenomenon**

- **Supplementary Figure S3A:** Leave-one-out sensitivity analysis

- **Supplementary Figure S3B:** Funnel plot

- **Supplementary Figure S3C:** Baujat plot

- **Supplementary Figure S3D:** Comprehensive influence diagnostics

**Supplementary Figure S4. Advanced diagnostics and sensitivity analysis for NOAF Prediction**

- **Supplementary Figure S4A:** Leave-one-out sensitivity analysis

- **Supplementary Figure S4B:** Funnel plot for publication bias

- **Supplementary Figure S4C:** Baujat plot and **Supplementary Figure S8D:** Comprehensive influence diagnostics
